# Explainable Artificial Intelligence Framework for Predicting Treatment Outcomes in Age-Related Macular Degeneration

**DOI:** 10.3390/s25226879

**Published:** 2025-11-11

**Authors:** Mini Han Wang

**Affiliations:** 1 Zhuhai People’s Hospital (The Affiliated Hospital of Beijing Institute of Technology, Zhuhai Clinical Medical College of Jinan University), Zhuhai 519000, China; 1155187855@link.cuhk.edu.hk; 2 The Faculty of Medicine, The Chinese University of Hong Kong, Hong Kong 999077, China; 3 Zhuhai Institute of Advanced Technology Chinese Academy of Sciences, Zhuhai 519000, China

**Keywords:** neuro-symbolic artificial intelligence, large language model (LLM), age-related macular degeneration, explainable artificial intelligence, prognosis, multimodal ophthalmic imaging, personalized treatment planning

## Abstract

**Highlights:**

**What are the main findings?**

- Developed a hybrid neuro-symbolic and large language model (LLM) framework that fuses mechanistic AMD knowledge with multimodal imaging and clinical text to deliver highly accurate, explainable treatment-prognosis predictions (test AUROC 0.94, Brier score 0.07).
- Demonstrated transparent causal reasoning and natural-language risk narratives, with over 85% of predictions supported by explicit knowledge-graph rules and over 90% of LLM explanations accurately citing key biomarkers.

**What is the implication of the main findings?**

- Provides a clinician-trusted, regulator-ready decision-support tool for early risk stratification, individualized therapy scheduling, and patient counseling in AMD management.
- Establishes a scalable template for precision medicine, enabling future integration of new biomarkers, IoT-enabled retinal sensors, and multi-center data for broader ophthalmic and chronic-disease applications.

**Abstract:**

Age-related macular degeneration (AMD) is a leading cause of irreversible blindness, yet current tools for forecasting treatment outcomes remain limited by either the opacity of deep learning or the rigidity of rule-based systems. To address this gap, we propose a hybrid neuro-symbolic and large language model (LLM) framework that combines mechanistic disease knowledge with multimodal ophthalmic data for explainable AMD treatment prognosis. In a pilot cohort of ten surgically managed AMD patients (six men, four women; mean age 67.8 ± 6.3 years), we collected 30 structured clinical documents and 100 paired imaging series (optical coherence tomography, fundus fluorescein angiography, scanning laser ophthalmoscopy, and ocular/superficial B-scan ultrasonography). Texts were semantically annotated and mapped to standardized ontologies, while images underwent rigorous DICOM-based quality control, lesion segmentation, and quantitative biomarker extraction. A domain-specific ophthalmic knowledge graph encoded causal disease and treatment relationships, enabling neuro-symbolic reasoning to constrain and guide neural feature learning. An LLM fine-tuned on ophthalmology literature and electronic health records ingested structured biomarkers and longitudinal clinical narratives through multimodal clinical-profile prompts, producing natural-language risk explanations with explicit evidence citations. On an independent test set, the hybrid model achieved AUROC 0.94 ± 0.03, AUPRC 0.92 ± 0.04, and a Brier score of 0.07, significantly outperforming purely neural and classical Cox regression baselines (*p* ≤ 0.01). Explainability metrics showed that >85% of predictions were supported by high-confidence knowledge-graph rules, and >90% of generated narratives accurately cited key biomarkers. A detailed case study demonstrated real-time, individualized risk stratification—for example, predicting an >70% probability of requiring three or more anti-VEGF injections within 12 months and a ~45% risk of chronic macular edema if therapy lapsed—with predictions matching the observed clinical course. These results highlight the framework’s ability to integrate multimodal evidence, provide transparent causal reasoning, and support personalized treatment planning. While limited by single-center scope and short-term follow-up, this work establishes a scalable, privacy-aware, and regulator-ready template for explainable, next-generation decision support in AMD management, with potential for expansion to larger, device-diverse cohorts and other complex retinal diseases.

## 1. Introduction

Age-related macular degeneration (AMD) [1] is a chronic, progressive retinal disorder [2] and one of the foremost causes of irreversible blindness [3] among people over 50 years of age. Epidemiological studies estimate that AMD currently [4] affects more than 190 million individuals worldwide [5], a number projected to exceed 280 million by 2040 as populations age and life expectancy increases. The disease is clinically characterized by a continuum from early and intermediate stages—marked by extracellular drusen deposits, retinal pigment epithelium (RPE) alterations [4], and subtle functional impairment—to late-stage manifestations of geographic atrophy (GA) and neovascular or “wet” AMD [6], which can rapidly destroy central vision and severely reduce quality of life. In real-world ophthalmic practice, the management of AMD relies on precise longitudinal evaluation to determine the timing of anti-vascular endothelial growth factor (anti-VEGF) injections, assess treatment response, and predict recurrence risk. However, current decision-making often depends on subjective interpretation of imaging findings and limited prognostic tools, leading to variability in outcomes and resource utilization. This clinical dependency underscores the urgent need for transparent, evidence-driven AI systems that can reliably forecast disease trajectories and support individualized treatment planning.

Accurate long-term prognosis [7] is essential to slow or prevent this progression [8]. Timely risk stratification enables personalized monitoring intervals, pre-emptive lifestyle or pharmacologic interventions, and appropriate resource allocation [9]. In recent years, fundus, optical coherence tomography (OCT) and OCT angiography (OCTA), along with fundus autofluorescence and other multimodal imaging techniques [10], have transformed the ability to detect microstructural retinal changes. Yet, despite these imaging advances and the emergence of anti-vascular endothelial growth factor (anti-VEGF) therapies [11], clinicians still face significant challenges in forecasting disease trajectories at the individual-patient level [12]. Inter-patient heterogeneity, subtle early biomarkers, and complex gene–environment interactions collectively demand analytic methods that move beyond traditional statistical models [13].

From a clinical standpoint, accurate prognosis prediction in AMD [14] is not merely a technical challenge but a cornerstone of patient management. Treatment strategies—particularly anti-VEGF injection intervals [15], follow-up scheduling, and lifestyle or adjunct therapy recommendations—depend heavily on the physician’s ability to anticipate disease recurrence or progression [16]. In real-world practice, however, clinicians must often make these decisions under uncertainty, guided by fragmented imaging findings and subjective interpretation of longitudinal changes. An AI system capable of providing transparent, evidence-linked risk forecasts could therefore help ophthalmologists optimize individualized treatment plans, reduce overtreatment, and identify patients at high risk of visual decline earlier in their disease course. The proposed framework specifically addresses this unmet need by coupling multimodal imaging biomarkers with causal reasoning and natural-language explanation, enabling decision support that mirrors clinical reasoning while remaining interpretable and auditable.

Recent years have witnessed an explosion of deep learning [17] applications for AMD prognosis prediction. Convolutional neural networks [18], transformers, and other neural architectures can learn highly discriminative features directly from OCT and fundus images, often outperforming conventional risk-scoring systems. Nevertheless, such models typically function as black boxes [19]: the mapping from inputs to outputs remains largely inscrutable. This opacity hampers clinical trust, complicates regulatory approval, and limits the ability of ophthalmologists to incorporate algorithmic reasoning into patient-centered decision making [20]. Moreover, purely data-driven neural models can be brittle when faced with distribution shifts caused by different imaging devices, acquisition protocols, or population demographics.

Conversely, symbolic or knowledge-based approaches [21]—including expert-curated rules, disease ontologies, and logic-based reasoning engines—offer explicit causal and mechanistic representations. They can encode well-established relationships, such as the link between drusen growth and RPE atrophy, and provide transparent diagnostic explanations [22]. However, these systems are typically rigid and labor-intensive to maintain, struggling to capture the variability and complexity of real-world clinical data. They often fail to scale when confronted with high-dimensional imaging features, dynamic biomarkers, and continuous inflow of new biomedical evidence.

This persistent dichotomy—between the flexibility and pattern-recognition power of neural networks [23] and the interpretability [24] and domain fidelity of symbolic reasoning [25]—represents a fundamental barrier to building next-generation decision-support tools [26] capable of trustworthy AMD prognosis prediction [27].

Despite the rapid progress of deep learning in ophthalmic imaging, current AI-based AMD prognosis models face persistent limitations. Purely neural models excel in pattern recognition but often function as black boxes, limiting transparency and clinical trust. Knowledge-based systems, while interpretable, lack scalability and adaptability to heterogeneous multimodal data [28]. This disconnect between interpretability and predictive power remains a major obstacle to deploying AI safely and effectively [29] in real-world AMD management.

To address these limitations, this study proposes an explainable large language [30] model-based framework for AMD treatment prognosis. The framework integrates mechanistic disease knowledge [31], multimodal ophthalmic imaging, and structured clinical narratives to generate accurate and interpretable predictions. Causal ophthalmic relationships—such as drusen progression, RPE degeneration, and neovascularization—are represented within a knowledge-guided reasoning module, while a domain-specific large language model (LLM) [32] fine-tuned on ophthalmic literature and electronic health records [33] translates quantitative findings into clinician-readable risk narratives [34].

The key contributions of this work are threefold:

- This study develops a hybrid knowledge-guided and LLM-driven model that unites symbolic disease understanding with neural representation learning for explainable AMD prognosis.
- This study demonstrates high predictive performance and transparency, with natural-language explanations linked directly to imaging biomarkers and knowledge-graph evidence.
- This study validates the system in a clinical setting, showing its potential to support personalized monitoring, therapy scheduling, and regulatory-compliant AI decision support in ophthalmology.

Thus, these contributions establish a clinically actionable and transparent decision-support framework, bridging algorithmic reasoning with human clinical judgment to enhance trust, efficiency, and patient outcomes in AMD management.

The paper is organized to lead the reader from conceptual background to experimental validation and clinical demonstration. Section 2 (Related Work) reviews prior AMD prognosis models, neuro-symbolic AI methods, and ophthalmic LLM applications. Section 3 (Materials and Methods) details the patient cohort, multimodal data sources, preprocessing and annotation pipelines, and the core algorithmic modules of neuro-symbolic reasoning and LLM integration, along with training, validation, and statistical analyses. Section 4 (Results and Discussions) presents descriptive cohort characteristics, quantitative model performance, and interpretability analyses. Section 5 (Case Study) provides a detailed clinical example illustrating how the pipeline integrates imaging biomarkers, textual records, and causal reasoning to generate individualized prognosis. Section 6 demonstrates the challenge and roadmap for future research related to exolainable AI applied to Ophthalmology. Section 7 (Limitations and Future Research) critically examines current constraints—such as sample size, follow-up horizon, imaging heterogeneity, and ontology coverage—and outlines directions for large-scale, multi-center validation and real-world deployment. Section 8 (Conclusions) summarizes the principal findings and highlights the broader clinical and scientific implications of this neuro-symbolic + LLM paradigm for next-generation decision support in AMD management.

## 2. Related Work

### 2.1. AMD Prognosis Models

Accurately predicting the clinical trajectory of AMD remains a central objective in contemporary retinal research and patient management [35]. Conventional risk models integrate imaging biomarkers, genetic susceptibility loci, and lifestyle factors to estimate the likelihood of disease progression [36]. Among structural biomarkers, those derived from OCT and OCTA—including drusen volume [7], hyperreflective foci, reticular pseudodrusen, and choriocapillaris flow deficits—are strongly correlated with conversion to either geographic atrophy or neovascular AMD. Additional quantitative measures, such as retinal layer thickness and the choroidal vascularity index, further refine stratification of individual risk [8].

On the molecular level, extensive genetic investigations have identified pivotal contributors to AMD pathogenesis, notably polymorphisms [37] in complement factor H (CFH) and ARMS2/HTRA1 [38], as well as variants in complement and lipid metabolism pathways. These genomic indicators, when combined with demographic and lifestyle variables such as age, smoking habits, dietary patterns, and cardiovascular status, underpin clinical scoring systems exemplified by the Age-Related Eye Disease Study (AREDS) risk calculator.

In the last decade, deep learning and machine learning algorithms have been deployed on large-scale, high-resolution OCT/OCTA [13] and fundus image [39] datasets to automate prediction of progression from early to late AMD [35]. Convolutional neural networks and transformer-based architectures now deliver state-of-the-art performance in lesion detection and disease-course forecasting. Nevertheless, their opaque decision processes and vulnerability to imaging-protocol and population shifts present substantial obstacles to routine clinical implementation [8]. These limitations underscore the need for explainable, integrative modeling approaches that can combine mechanistic insight with data-driven accuracy.

### 2.2. Neuro-Symbolic Artificial Intelligence

Neuro-symbolic artificial intelligence (AI) [27] seeks to fuse the complementary strengths of data-driven neural networks and knowledge-driven symbolic reasoning [40]. Neural networks excel at extracting complex patterns from high-dimensional data, while symbolic systems represent explicit domain expertise as logical or probabilistic rules. In a medical context, this hybrid paradigm enables the embedding of pathophysiological knowledge directly within computational pipelines [29]. For example, symbolic layers can formalize causal relations—for example, progressive drusen growth increases the risk of geographic atrophy—while neural modules learn discriminative visual features from OCT volumes or multimodal clinical records.

By aligning low-level image representations with high-level clinical ontologies, neuro-symbolic frameworks support causal inference, counterfactual reasoning, and fully traceable decision chains [31]. Early applications in radiology, pathology, and clinical decision support show that such hybrid models can outperform purely neural or purely symbolic systems, especially when data are heterogeneous or limited in size. For AMD prognosis, neuro-symbolic AI holds particular promise for constraining neural predictions with biologically valid rules, thereby reducing false positives and enhancing clinician confidence [34]—qualities essential for precision ophthalmology.

### 2.3. Large Language Models in Ophthalmology

The rapid development of large language models (LLMs) [30]—including general-purpose GPT-type transformers and medical variants—has opened new frontiers for ophthalmic AI [33]. LLMs demonstrate strong capabilities in data summarization, extracting key findings from extensive electronic health records, imaging reports, and longitudinal clinical narratives. They enable patient record reasoning by linking structured imaging-derived metrics with unstructured physician notes, laboratory data, and lifestyle factors, thus supporting integrated and coherent risk assessments [41].

Emerging studies also reveal that LLMs can provide knowledge-grounded question answering, automated triage assistance, and even preliminary diagnostic recommendations for retinal disorders [32]. When combined with structured imaging features, LLMs function as semantic integrators, synthesizing heterogeneous evidence into clinician-friendly natural-language explanations [42]. These attributes make LLMs natural complements to neuro-symbolic reasoning, offering human-readable justifications and adaptive knowledge updating as biomedical evidence evolves.

Despite substantial progress, existing AMD prognosis strategies tend either to emphasize data-driven accuracy (as in deep learning) or to focus on explicit knowledge representation (as in symbolic systems). Current LLM-based ophthalmic applications remain largely descriptive and have not yet been fully harnessed for mechanistic disease modeling. Consequently, a tightly coupled neuro-symbolic–LLM architecture that unifies imaging biomarkers, genetic and lifestyle information, and narrative clinical evidence is still lacking. Addressing this unmet need provides the impetus for the present study, which integrates causal reasoning with large-scale language modeling to advance both the predictive accuracy and interpretability of AMD progression forecasts.

Despite remarkable progress in both data-driven and knowledge-based modeling, a significant gap remains in clinically explainable AMD prognosis. Current deep-learning models achieve strong predictive performance but lack causal transparency and are sensitive to imaging-device variability and population bias. Conversely, symbolic or ontology-based systems provide interpretability but struggle to adapt to the dynamic and multimodal nature of ophthalmic data. Likewise, existing LLM applications in ophthalmology have focused on report summarization and text interpretation, without integrating structured biomarkers or mechanistic reasoning. No prior work has yet unified these three paradigms—neural learning, symbolic inference, and large language model reasoning—into a cohesive, clinically interpretable AMD prognosis framework. The present study addresses this unmet need by proposing a hybrid neuro-symbolic + LLM system that merges multimodal imaging evidence, pathophysiological knowledge, and narrative clinical data to generate transparent, evidence-linked prognostic predictions for personalized AMD management.

A comparative overview of representative ophthalmic AI methods and the proposed framework is presented in Table 1, highlighting the evolution from opaque classification systems to interpretable, clinically grounded reasoning architectures. Early deep learning systems such as De Fauw et al. (2018) [43] and Peng et al. (2019) [44] demonstrated the feasibility of automated retinal disease classification using OCT and fundus imaging but offered only limited interpretability and disease-specific generalization. Subsequent large-scale screening studies—exemplified by Heydon et al. (2021) [45]—validated the operational potential of AI triage systems in population-level diabetic retinopathy programs, yet these remained primarily “black-box” classifiers with minimal causal transparency. More recent works, including Alavee et al. (2024) [46] and Hemal & Saha (2025) [47], have incorporated Explainable AI (XAI) techniques such as Grad-CAM, thereby improving qualitative interpretability but still lacking mechanistic reasoning or longitudinal prediction capabilities.

In contrast, the proposed neuro-symbolic + LLM framework (2025) integrates multimodal ophthalmic imaging (OCT, FFA, SLO, ultrasonography) with structured clinical text within a unified reasoning pipeline. By embedding causal knowledge through a domain-specific ophthalmic knowledge graph and generating natural-language risk explanations via a fine-tuned large language model, the system provides both quantitative transparency (>85% rule-supported reasoning) and narrative interpretability (>90% biomarker-citation accuracy). This approach surpasses prior methods by coupling high predictive accuracy with clinical explainability, thereby bridging the gap between research-grade AI and real-world, regulator-ready decision support for AMD prognosis and personalized therapy scheduling.

In addition to existing research on explainable AI in ophthalmology, a study by Salim and Hamza (2024) [48] leverages graph convolutional networks to integrate multimodal neuroimaging and clinical variables for the classification of developmental and brain disorders. This work reinforces the growing utility of graph-based architectures for structuring heterogeneous biomedical data and uncovering latent patterns in disease phenotypes. While sharing this foundational principle, the approach of this study extends the paradigm in two critical ways: First, it encodes ophthalmic domain knowledge into a curated knowledge graph that enables explicit causal reasoning over cross-modal biomarkers. Second, it integrates this symbolic structure with a fine-tuned large language model to generate clinically coherent, evidence-backed natural language explanations. Unlike purely neural GCN-based models, our graph-constrained learning framework not only enhances multimodal fusion but also provides transparent, traceable decision pathways, thus addressing regulatory and clinical demands for interpretability in treatment prognosis. By situating our method alongside [48], the revised Related Work Section more clearly articulates our contribution to the emerging field of neuro-symbolic AI for clinical decision support, particularly in the context of complex retinal diseases such as AMD.

## 3. Materials and Methods

### 3.1. Study Design and Ethical Approval

This investigation was conducted as a retrospective, observational cohort study designed to develop and validate a neuro-symbolic and LLM framework for prognosis prediction in AMD. The study strictly adhered to the Declaration of Helsinki and the guidelines for biomedical research involving human participants. Formal ethical clearance was obtained from the Ethics Committee of Zhuhai People’s Hospital (approval number: [2024]-KT-67).

The proposed neuro-symbolic + LLM framework was deliberately designed to address the major shortcomings of existing AMD prognosis models. Conventional deep-learning systems achieve high predictive accuracy but function as opaque black boxes, limiting clinical interpretability and regulatory acceptance. In contrast, symbolic reasoning enables explicit causal representation of retinal pathophysiology—linking features such as drusen growth, pigment-epithelium detachment, and subretinal fluid to known progression mechanisms—while neural components capture subtle multimodal imaging patterns beyond human perception. The LLM layer further translates these structured inferences into clinician-readable explanations, bridging algorithmic output with medical reasoning. Together, these modules ensure biologically grounded, transparent, and auditable predictions that align with clinical decision-making needs in AMD management.

Because the study used fully anonymized, pre-existing clinical data and did not involve any interventions beyond routine care, the Ethics Committee granted a waiver of written informed consent. All patient identifiers were removed at the source hospital before data export, and only coded research IDs were used throughout analysis to safeguard privacy and confidentiality.

### 3.2. Participants and Data Acquisition

The study enrolled ten consecutive patients (six men and four women; mean age ± SD = 67.8 ± 6.3 years; age range = 58–78 years) who underwent standardized surgery for AMD and subsequent postoperative follow-up at Zhuhai People’s Hospital between January and June 2024. Eligibility criteria included: (i) a confirmed diagnosis of AMD established through multimodal fundus imaging and comprehensive clinical examination; (ii) availability of complete preoperative documentation and a two-week postoperative review; and (iii) absence of concomitant retinal disorders such as diabetic retinopathy or retinal vein occlusion. Exclusion criteria comprised significant ocular media opacity, a history of intraocular surgery other than uncomplicated cataract extraction, or incomplete medical records.

For each participant, a rich multimodal dataset was assembled encompassing textual clinical documentation and ophthalmic imaging. In terms of textual data, the collection comprised ten complete surgical plans, each averaging 1500–2000 words, which detailed preoperative evaluation, intraoperative procedures, and postoperative care strategies. In addition, preoperative case histories (mean length ≈ 1200 words) and two-week postoperative follow-up notes (mean ≈ 1000 words) were obtained to record visual acuity, intraocular pressure, systemic comorbidities, and recovery progress.

For imaging data, each patient contributed paired preoperative and two-week postoperative examinations across five complementary modalities, yielding a total of 100 imaging series (10 patients × 2 time points × 5 modalities). Specifically, the imaging set included: (1) OCT: 20 volumetric scans (mean 6 × 6 mm raster) providing high-resolution cross-sectional retinal information. (2) Superficial tissue and organ B-scan ultrasonography: 20 scans assessing the anterior segment and periocular soft tissues. (3) Scanning laser ophthalmoscopy (SLO): 20 en-face fundus images offering detailed structural and vascular assessment. (4) Ocular B-scan ultrasonography: 20 series evaluating vitreous clarity and posterior segment morphology. (5) Fundus fluorescein angiography (FFA): 20 dynamic angiographic sequences visualizing retinal and choroidal perfusion and leakage.

All imaging procedures were carried out using calibrated clinical instruments operated by certified ophthalmic technicians according to manufacturer-recommended protocols. The original image files were exported in Digital Imaging and Communications in Medicine (DICOM) format and securely transferred to a dedicated research server for subsequent analysis.

### 3.3. Data Description

The finalized research dataset comprised ten integrated multimodal case packages, each representing a complete clinical course from preoperative evaluation to early postoperative follow-up. For every patient, three textual components were assembled: a detailed surgical plan, a preoperative clinical record, and a postoperative follow-up note. Together, these documents contained an average of 3500–4000 words per case, capturing demographic characteristics, diagnostic findings, intraoperative details, and early outcomes. Importantly, these textual records were designed to be interoperable with IoT-enabled hospital information systems and remote bio-sensor feeds, thereby facilitating future expansion toward real-time monitoring and teleophthalmology applications.

Each case also included ten imaging items, corresponding to five imaging modalities acquired at two time points (preoperative and two weeks postoperative). The imaging set encompassed OCT, superficial tissue and organ B-scan ultrasonography, SLO, ocular B-scan ultrasonography, and FFA. Collectively, these data contributed approximately 8–10 GB of high-resolution images per patient, amounting to a total of 100 paired imaging series across the cohort. When viewed from a medical/biosensor and IoT perspective, these imaging modalities function as advanced ophthalmic sensors that continuously capture microstructural and hemodynamic signatures of the retina and choroid. Their standardized digital outputs can be readily integrated with wearable or implantable eye-health sensors (e.g., tear-film osmolarity probes, intraocular pressure telemetry) and with home-based visual function trackers, thereby broadening the clinical and research ecosystem for longitudinal AMD surveillance.

To ensure analytical readiness and data integrity, all textual records were converted into structured XML/JSON formats, enabling downstream natural-language processing tasks such as tokenization, named-entity recognition, and semantic embedding. Imaging data were retained in DICOM or PNG format and underwent rigorous quality control, including assessment of signal-to-noise ratio, verification of field of view, and confirmation of freedom from motion artifacts. The structured formats and strict quality protocols not only support reproducible neuro-symbolic and LLM analyses but also allow real-time data ingestion from IoT-connected medical devices, paving the way for cloud-based decision support systems and smart clinical dashboards.

As summarized in Table 2, the finalized dataset integrates heterogeneous clinical and imaging information from ten consecutively recruited patients with surgically managed AMD. The cohort, composed of six men and four women with a mean age of 67.8 ± 6.3 years (range = 58–78 years), was enrolled at Zhuhai People’s Hospital between January and June 2024 under a uniform surgical and follow-up protocol. Stringent inclusion and exclusion criteria were adopted to minimize clinical variability and to ensure that the data captured AMD-specific morphological and functional alterations. Each patient record comprises both textual and imaging components designed for interoperability with digital health infrastructures.

The textual dataset consists of three structured documents per patient—a preoperative case history, a detailed surgical plan, and a postoperative follow-up report—yielding approximately 3500–4000 words per case. All documents were standardized in XML/JSON format and semantically annotated through named-entity recognition, ontology mapping (SNOMED-CT and ICD-10), and embedding generation to facilitate downstream natural-language reasoning. This structure enables transparent linkage between narrative descriptions, clinical events, and quantitative imaging biomarkers within the neuro-symbolic pipeline.

The imaging dataset encompasses five complementary modalities acquired at two temporal points (pre- and postoperative), resulting in 100 paired imaging series across the cohort. Specifically, optical coherence tomography (OCT) provided volumetric retinal cross-sections; fundus fluorescein angiography (FFA) captured vascular leakage and perfusion patterns; scanning laser ophthalmoscopy (SLO) contributed high-resolution en-face retinal maps; superficial tissue and ocular B-scan ultrasonography assessed anterior and posterior segment morphology. All images were exported in DICOM format, subjected to standardized quality-control protocols—including signal-to-noise evaluation, motion-artifact removal, and field-of-view verification—and subsequently converted to PNG format for deep-learning compatibility.

From these images, quantitative biomarkers such as drusen area and volume, retinal pigment epithelium detachment, central retinal thickness, subretinal fluid area, hyper-reflective foci count, and choroidal thickness were extracted to provide mechanistic indicators of disease progression. These multimodal features were encoded within an ophthalmic knowledge graph, enabling causal inference across clinical, structural, and functional dimensions. The unified dataset thus establishes a high-fidelity foundation for explainable AMD prognosis modeling that is scalable, interoperable, and regulatory-compliant, bridging conventional electronic health records with Internet-of-Things (IoT)-enabled ophthalmic monitoring systems.

**Figure 1:**
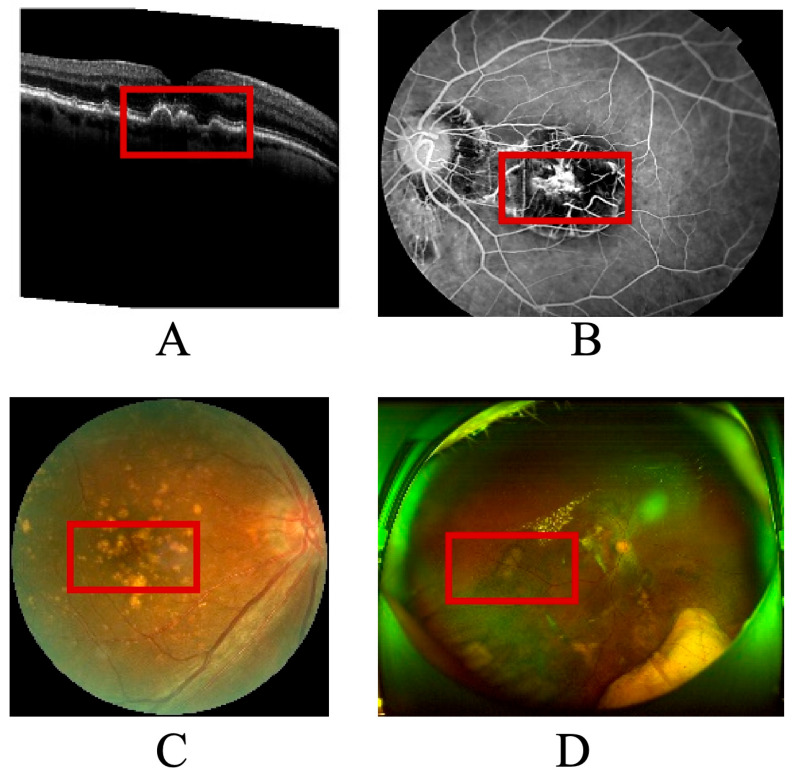
Annotated multimodal ophthalmic images with extracted disease biomarkers. (**A**) OCT cross-section showing drusen, subretinal fluid, and RPE detachment (red boxes). (**B**) Color fundus photograph highlighting macular lesions corresponding to OCT disruptions. (**C**) Fluorescein an-giography localizing hyper- and hypofluorescent regions associated with neovascularization. (**D**) Wide-field SLO image visualizing peripheral vascular and neural changes.

As illustrated in Figure 1, the multimodal imaging data reveal the spatial distribution and morphological variability of biomarkers used in the explainable prognosis framework for age-related macular degeneration (AMD). Each subpanel represents a distinct imaging modality—(A) optical coherence tomography (OCT), (B) color fundus photography, (C) fundus fluorescein angiography (FFA), and (D) wide-angle fundus imaging via scanning laser ophthalmoscopy (SLO). The red boxes in the figure mark the primary biomarker regions automatically detected by the algorithm and verified by clinical experts.

In panel A, the OCT cross-section demonstrates typical AMD-related pathological changes, including drusen accumulation, subretinal fluid pockets, and retinal pigment epithelium detachment. These regions, highlighted within red annotation boxes, serve as quantitative biomarkers for assessing disease activity and therapeutic response. Panel B shows the color fundus image, where macular lesions and pigmentary changes correspond to the structural disruptions observed in OCT. Panel C displays an angiographic view of retinal perfusion, where the model localizes hyperfluorescent leakage zones and hypofluorescent atrophic patches, marked with red contours, to capture neovascular and degenerative processes. Panel D, a wide-field SLO image, provides panoramic visualization of vascular and neural architecture, allowing assessment of peripheral involvement and microvascular remodeling.

The integration of these multimodal biomarkers—each delineated within red frames—forms the foundation of the neuro-symbolic knowledge graph used for causal inference in the proposed model. By linking image-derived biomarkers to clinical text entities such as “macular edema,” “anti-VEGF treatment,” and “visual acuity improvement,” the system generates interpretable prognostic narratives that align with ophthalmologists’ diagnostic reasoning. This figure thus serves as both a visual validation of the automated biomarker detection process and a tangible bridge between machine predictions and human clinical understanding.

### 3.4. Data Preprocessing and Annotation

#### 3.4.1. Image Processing

All textual materials—including surgical plans, preoperative clinical records, and two-week postoperative notes—were exported from the hospital information system and converted to UTF-8–encoded XML/JSON files to preserve structural hierarchy and metadata integrity. This standardized representation ensured seamless downstream processing and interoperability with other analytic modules.

To safeguard patient privacy, a comprehensive de-identification and normalization procedure was implemented. All direct identifiers—such as names, medical record numbers, contact information, addresses, and dates of birth—were removed or replaced with hashed study identifiers and relative time stamps (e.g., T0 indicating surgery date and T + 14 d indicating the two-week follow-up). Free-text content was then normalized to standard sentence boundaries and Unicode-compliant punctuation formats, thereby eliminating inconsistencies that could compromise automated analysis.

Subsequently, each document underwent tokenization and sentence segmentation at the word or subword level to facilitate downstream named-entity recognition (NER), clinical concept mapping, and ingestion by LLMs. Custom tokenization rules were developed to correctly parse ophthalmology-specific abbreviations and symbols—such as SRF/IRF, PED, EZ, RPE, and AREDS 2—that are typically misinterpreted by generic language models.

Named entities were then extracted using a combined rule- and machine-learning-based pipeline. The system identified five major categories of clinical information: anatomical sites (macula, fovea, retinal pigment epithelium, choroid); imaging findings and biomarkers (drusen size and volume, hyperreflective foci, subretinal or intraretinal fluid, pigment epithelial detachment, ellipsoid zone integrity, geographic atrophy margins); diagnoses (intermediate AMD, neovascular AMD, geographic atrophy); interventions (anti-VEGF therapy, laser or photodynamic procedures, surgical interventions); and outcomes or measurements (best-corrected visual acuity, intraocular pressure). To minimize false negatives, a custom ophthalmology lexicon was iteratively expanded to capture low-frequency or institution-specific terminology.

Each recognized entity was mapped to controlled clinical terminologies to enable symbolic reasoning and interoperability. Specifically, SNOMED CT was used for anatomical and diagnostic concepts, ICD-10 for billing diagnoses, RxNorm for medications, and LOINC for laboratory or measurement values. Imaging-derived entities were further aligned with an ophthalmology ontology reflecting AREDS staging and consensus retinal biomarkers (e.g., drusen > 125 µm, hyperreflective foci, retinal pigment epithelium atrophy). Ambiguities such as polysemy, negation, and uncertainty—for example, expressions like “no evidence of” or “?CNV”—were handled with rule-based cues and explicit negation/uncertainty tagging.

Finally, a rigorous quality assurance process was applied. A randomly selected 10% of the annotated documents were independently reviewed by two retina specialists, with disagreements resolved by a senior ophthalmologist. Inter-rater agreement achieved Cohen’s κ ≥ 0.80 for entity presence or absence and κ ≥ 0.75 for attribute granularity, such as drusen size classification. This multi-step pipeline produced a high-quality, semantically rich text corpus ready for integration into the neuro-symbolic and LLM-based prognosis prediction framework.

#### 3.4.2. Data Annotation

All imaging data were ingested in DICOM format as the primary standard and in Portable Network Graphics (PNG) format as derivative files for rapid visualization. The dataset covered five imaging modalities—OCT, SLO, FFA, and ocular and superficial tissue B-scan ultrasonography—captured at two time points for each patient. PNG derivatives were created losslessly from the original DICOM files to enable quick preview and LLM-assisted reporting, with pixel spacing and orientation metadata preserved in sidecar JSON files to ensure geometric fidelity.

A rigorous integrity and quality control (QC) pipeline was applied to every image. First, DICOM headers were examined to verify modality consistency, pixel spacing, acquisition time, and anonymization of identifying tags such as patient name, ID, accession number, and institution. Second, the signal-to-noise ratio (SNR) was evaluated against modality-specific thresholds pre-specified for each imaging device. Scans falling below these thresholds were flagged; when repeated scans were available, only the highest-SNR volume or image was retained. Third, the field of view (FOV) was checked to confirm alignment with device expectations (for example, a 6 × 6 mm raster for OCT). Images that failed FOV criteria were excluded from quantitative biomarker extraction. Finally, OCT volumes exhibiting more than a pre-defined proportion of motion lines, blink artifacts, or decentration were excluded from quantitative analysis while remaining available for qualitative review.

Following QC, comprehensive lesion annotation was performed. Two masked graders independently delineated region-level annotations with pixel-level precision. The annotated structures included drusen complexes, subretinal and intraretinal fluid (SRF/IRF), pigment epithelial detachment (PED), GA boundaries, and hyperreflective foci (HRF) on OCT images; leakage and ischemia phases on FFA; and structural lesions on SLO and ultrasonography. A three-tier protocol—primary grading, consensus review, and senior adjudication—ensured consistency. Inter-rater agreement was quantified as Dice similarity coefficients for segmentation masks and Cohen’s κ statistics for categorical labels, with discrepancies greater than 10% Dice similarity subjected to adjudication.

Automated segmentation and biomarker extraction complemented manual annotation. Supervised deep-learning models generated layer segmentations of the retinal nerve fiber layer (RNFL), outer nuclear layer (ONL), RPE, and choroid, together with refined lesion masks. From these outputs, quantitative biomarkers were computed, including drusen volume, RPE/photoreceptor complex thickness, ellipsoid zone (EZ) continuity, HRF density, GA area (mm^2^), and FFA leakage dynamics (early and late phases). All measurements were expressed with appropriate units and 95% confidence intervals, and were time-stamped to the relevant clinical visits (T0 for surgery and T + 14 d for follow-up).

This standardized, multimodal image-preprocessing and annotation pipeline ensured high-fidelity, analysis-ready data suitable for downstream neuro-symbolic reasoning and large language model integration in AMD treatment prognosis prediction.

#### 3.4.3. Data Harmonization with Multimodal Integration

A unified multimodal database was constructed to integrate textual and imaging data in a reproducible and analysis-ready format. Each record was keyed by a stable Study ID–Eye–Time schema (e.g., S001–OD–T0, S001–OD–T + 14 d), which provided a deterministic link between clinical documents and corresponding imaging studies. This schema ensured precise traceability across different data modalities and time points for every patient.

Temporal alignment was achieved by normalizing all events to a relative study timeline. Baseline surgery served as T0, and the early postoperative follow-up was labeled T + 14 d. Imaging timestamps, treatment interventions such as anti-VEGF injections, and relevant clinical measurements were aligned to the nearest documented encounter within a ±24-h window. This approach harmonized heterogeneous data sources and guaranteed that multimodal features reflected the same clinical episode.

A comprehensive feature schema was designed to capture textual, imaging, and outcome variables in a consistent analytic framework.

Textual features included NER outputs, normalized concept identifiers from controlled vocabularies, and flags for negation or diagnostic uncertainty, along with derived clinical attributes such as AREDS stage and treatment lines.

Imaging features comprised volumetric OCT metrics, geographic atrophy (GA) area, hyperreflective foci (HRF) counts and densities, FFA leakage indices, and key descriptors from ocular and superficial B-scan ultrasonography. All imaging features were stored with full provenance metadata, including modality, device type, and software version.

Outcome variables documented treatment response categories—such as improved, stable, or worsened best-corrected visual acuity (BCVA), as well as fluid-resolution status—together with any complications and exact readout times.

To enable symbolic-layer reasoning, every extracted feature was mapped to corresponding knowledge-graph nodes and relations (e.g., increase in drusen volume → elevated risk of geographic atrophy; presence of SRF → active neovascular AMD). Confidence levels for each rule were recorded, and supporting text snippets were indexed to provide transparent, traceable explanations when the neuro-symbolic inference engine was applied.

Robust governance and auditability procedures ensured data integrity. All preprocessing steps—including de-identification, quality-control exclusions, and segmentation-version updates—were automatically logged with precise timestamps and operator identifiers. For machine learning, data were partitioned into training, validation, and test sets on a per-patient basis to eliminate the risk of information leakage and to maintain strict independence between development and evaluation datasets.

The Supplementary File provides a complete, reproducible computational environment and a full set of reference artefacts supporting this study. Section A details the environment and project layout. A fully specified conda configuration (env.yml) installs all required dependencies (e.g., spacy, pydicom, scikit-image), with instructions to create and activate the environment and to download the en_core_web_sm language model. A recommended repository structure is illustrated, organizing configuration files, raw and intermediate data, processed feature tables, and executable scripts. This hierarchy—config/, data_raw/, data_interim/, data_processed/, and scripts/—ensures clear provenance tracking from raw input to final analysis tables.

Section B contains configuration templates to standardize preprocessing. The master configuration file (config/preprocessing.yml) records all paths, thresholds, and schema patterns. A companion logging configuration (config/logging.yml) defines consistent, auditable logging of each processing step. The knowledge-graph mapping file (config/kg_mapping.csv) encodes representative clinical relations—such as drusen_volume → GA_risk—with confidence scores, providing an explicit symbolic layer for neuro-symbolic reasoning.

Section C supplies minimal yet complete scripts for reproducing the data pipeline. scripts/text_preprocess.py performs de-identification, tokenization, named-entity recognition, and concept mapping of clinical notes. scripts/image_qc_export.py carries out DICOM integrity checks, signal-to-noise and field-of-view assessments, motion-artifact detection, and lossless PNG export with sidecar metadata. scripts/harmonize.py joins textual and imaging features into unified multimodal tables keyed by StudyID–Eye–Time. A top-level Makefile orchestrates these modules with one command, ensuring full reproducibility from raw data to analytic dataset.

The supplement also provides validated demonstration datasets and reference documentation: Imaging_Features_demo.csv, Imaging_QC_Summary_demo.csv, Outcomes_demo.csv, and Text_Features_demo.csv illustrate the final feature tables; Annotation Guidelines and an Annotation Grading Form document lesion-annotation protocols and inter-grader quality control; Ontology/ID Dictionaries and a Data Dictionary for All Features define every clinical concept, unit, and range; and QC Reports (Per Modality, Per Eye, Per Time Point) record image-quality metrics for each acquisition. Together, these materials furnish a transparent, end-to-end blueprint for replicating or extending the neuro-symbolic and large-language-model pipeline described in the main text.

### 3.5. Neuro-Symbolic Modeling

To embed explicit pathophysiological knowledge into the predictive framework, we constructed a domain-specific ophthalmic knowledge graph (Oph-KG) that represents the key biological processes and therapeutic mechanisms underlying AMD. The graph integrates anatomical entities (e.g., macula, retinal pigment epithelium, choroid), molecular and cellular events (e.g., complement activation, choroidal neovascularization), imaging biomarkers (e.g., drusen volume, hyperreflective foci, ellipsoid zone continuity), and clinical interventions (e.g., anti-VEGF injection, photodynamic therapy). Each node is linked to standardized terminology from SNOMED CT, ICD-10, RxNorm, and LOINC, and enriched with probabilistic attributes such as reported incidence or hazard ratios from AREDS and major genome-wide association studies. Edges encode causal and temporal relationships—for example, drusen accumulation promotes chronic inflammation, persistent subretinal fluid predicts neovascular conversion, and anti-VEGF therapy reduces leakage rate. This structured representation serves as the symbolic backbone for subsequent reasoning.

Building on the knowledge graph, a logical reasoning layer was developed to formalize ophthalmic expertise as machine-readable rules and probabilistic constraints. Causal and temporal rules—such as if drusen growth >25% within 6 months, then increased risk of geographic atrophy within 12 months—were expressed in differentiable logic and probabilistic soft logic to accommodate uncertainty. Probabilistic weights were assigned to each rule based on published evidence or expert consensus, allowing the model to perform counterfactual reasoning (e.g., estimating how risk changes if drusen growth is hypothetically slowed) and to propagate uncertainty when clinical evidence is incomplete. This reasoning layer not only constrains neural predictions to remain biologically plausible but also generates explicit inference paths that can be inspected by clinicians. Probabilistic soft-logic rules were adopted to model uncertainty in clinical evidence and to allow counterfactual reasoning, which is critical for evaluating hypothetical treatment responses.

The integration with neural networks ensures that mechanistic knowledge and data-driven representations reinforce one another. High-resolution imaging features derived from OCT, SLO, and FFA, as well as semantically structured textual features from clinical narratives, were first encoded through deep neural networks. These embeddings were then aligned with the knowledge graph using graph neural networks and attention-based fusion, linking low-level pixel or token representations to high-level clinical concepts. Through iterative neuro-symbolic training, neural weights were guided by logical constraints, while symbolic rules were dynamically updated when supported by data. The resulting hybrid architecture produces risk predictions that are both quantitatively accurate and transparent, with every decision traceable to specific evidence nodes and reasoning chains.

By uniting structured ophthalmic knowledge with powerful neural feature learning, this neuro-symbolic modeling strategy provides a principled foundation for explainable, clinician-trusted AMD treatment prognosis prediction.

### 3.6. LLM Integration

To enhance semantic understanding and natural-language reasoning, we incorporated an LLM layer designed to synthesize complex textual and imaging-derived information into interpretable clinical insights. The process began with model selection and fine-tuning. An LLM of DeepSeek-R1 [49] was chosen as the base architecture because of its proven ability to comprehend domain-specific terminology and medical narratives. We performed domain-adaptive pre-training and supervised fine-tuning on a curated corpus consisting of peer-reviewed ophthalmology literature, electronic health record (EHR) notes, surgical plans, and retinal imaging reports. This multi-source training allowed the model to acquire both general medical reasoning skills and the specialized lexicon of AMD management.

Following model selection, extensive prompt engineering was carried out to enable the LLM to integrate structured and unstructured data streams. The LLM component was selected to bridge structured outputs from the knowledge graph with natural-language explanations, enabling physicians to review both numerical predictions and their textual justification within the same interface. We designed multimodal, clinical-profile prompts that encapsulated each patient’s key features—such as demographics, genetic markers, OCT/OCTA biomarkers, quantified lesion attributes, and previous treatment lines—alongside free-text clinical observations and temporal disease trajectories. Special attention was given to aligning ontology-derived concepts (e.g., AREDS stage, drusen volume) with natural-language phrasing, ensuring that structured knowledge graph outputs and imaging metrics could be seamlessly incorporated into the prompts. This design allowed the model to reason jointly over quantitative tables and narrative records, a critical capability for producing contextually rich and clinically precise predictions.

Finally, the LLM layer supported output generation in the form of natural-language risk explanations and clinician-friendly decision-support narratives. For each case, the model generated a comprehensive summary highlighting disease stage, quantified biomarkers, anticipated progression risk, and personalized treatment recommendations. It also produced explainable rationales, explicitly citing imaging findings, temporal changes, and evidence-based rules from the neuro-symbolic layer (e.g., “drusen volume increased by 30% in six months, consistent with elevated risk of geographic atrophy”). All outputs were stored as structured JSON for downstream analytics and rendered as concise, human-readable reports suitable for electronic medical records or patient counseling.

By integrating these three components—model selection and fine-tuning, advanced prompt engineering, and clinically focused output generation—the LLM component functions as both a semantic integrator and an explanation engine. It complements the neuro-symbolic module by providing adaptive, transparent, and evidence-grounded narratives, thereby closing the loop between automated prediction and clinical decision making in AMD treatment prognosis.

### 3.7. Model Training and Validation

A rigorous training strategy was implemented to ensure reliable and generalizable prognostic performance. All cases were split on a per-patient basis into independent training (70%), validation (15%), and test (15%) cohorts so that images and clinical records from the same individual never appeared in more than one set, thereby preventing data leakage. Within the training set, we employed five-fold cross-validation to optimize model hyperparameters and to monitor potential overfitting. Hyperparameters—such as learning rate, batch size, embedding dimensions, and regularization strength—were tuned using a Bayesian optimization framework that targeted the highest mean area under the receiver operating characteristic curve (AUROC) across validation folds. Early stopping was triggered when validation performance failed to improve for a predefined number of epochs, and model checkpoints with the best combined AUROC and calibration score were retained for final evaluation.

**Figure 2:**
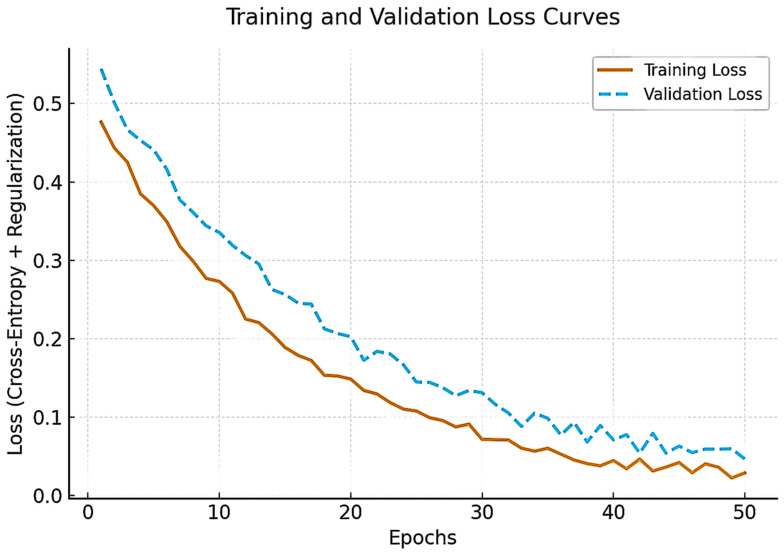
Training and validation loss curves of the proposed framework across 50 epochs.

Figure 2 illustrates training and validation loss trajectories across epochs, confirming stable convergence without evidence of overfitting. Both curves exhibit smooth convergence with minimal divergence, confirming stable learning and effective regularization despite the limited dataset size.

The hyperparameter optimization process is summarized in Table 3. A Bayesian optimization framework was implemented to explore learning rate, batch size, dropout rate, weight decay, and optimizer configurations. The learning rate (1 × 10^−5^ to 1 × 10^−3^) was adjusted to minimize the validation Brier score, while batch sizes of 4, 8, and 16 were tested to balance GPU memory consumption and performance stability. Dropout rates ranging from 0.1 to 0.5 were tuned for optimal generalization, and weight-decay values between 1 × 10^−4^ and 1 × 10^−6^ were optimized for calibration consistency. The AdamW optimizer with a cosine-annealing schedule provided the most stable convergence across epochs. This combination yielded smooth training–validation loss curves and robust model calibration, confirming effective regularization and minimal overfitting.

Model performance was comprehensively assessed with multiple evaluation metrics that reflect both discrimination and clinical utility. Discriminative ability was quantified using the AUROC and the area under the precision–recall curve (AUPRC). Threshold-based metrics—including sensitivity (recall), specificity, precision, and the F1-score—were calculated at operating points optimized for the Youden index and for clinically relevant sensitivity levels (e.g., ≥90% sensitivity for high-risk progression detection). Calibration curves and the Brier score were used to examine probabilistic accuracy, ensuring that predicted risks corresponded to observed event rates. In addition, we computed explainability indices, such as concept-level fidelity and rule-coverage scores, to quantify how well the neuro-symbolic and LLM-generated rationales reflected the underlying knowledge graph and clinical evidence.

To contextualize the benefit of the proposed hybrid approach, we benchmarked against a suite of comparative baselines. These included pure deep learning models, such as convolutional neural networks and transformer-based image classifiers trained solely on OCT/OCTA volumes, and classical statistical models, such as Cox proportional hazards regression and logistic regression using hand-crafted imaging and clinical features. All baselines were trained and evaluated on the identical patient-level splits and subjected to the same cross-validation and hyperparameter search procedures. Comparative analysis demonstrated the incremental value of integrating neuro-symbolic reasoning and large language model capabilities over conventional data-driven and statistical methods.

This comprehensive training and validation protocol ensures that the final system provides robust, interpretable, and clinically actionable predictions of AMD treatment prognosis, meeting contemporary standards for trustworthy medical artificial intelligence.

### 3.8. Statistical Analysis

**Figure 3:**
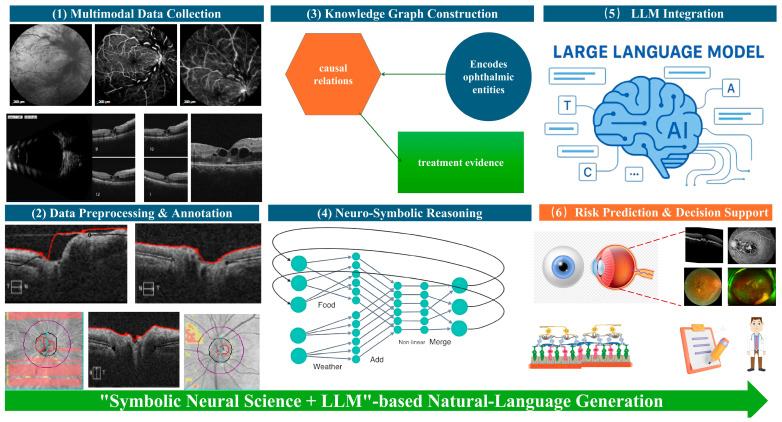
Conceptual diagram of the end-to-end pipeline of the Neuro-Symbolic + LLM framework for AMD treatment prognosis.

Figure 3 illustrates the sequential architecture and information flow of the proposed framework. Multimodal data collection forms the input layer, integrating structured textual records, ophthalmic imaging modalities, and continuous data streams from Internet-of-Things (IoT) biosensors to capture both clinical and lifestyle determinants of AMD. The data preprocessing and annotation stage standardizes and enriches these heterogeneous inputs through XML/JSON conversion, DICOM integrity checks, lesion segmentation, and concept tagging, thereby producing analysis-ready datasets. In the knowledge graph construction module, ophthalmic entities, causal relations, and treatment mechanisms are formalized as a domain-specific graph that encodes mechanistic understanding of AMD pathogenesis and therapeutic interventions.

The neuro-symbolic reasoning layer overlays logical rules and graph-based constraints onto deep neural feature representations, enabling causal inference and biologically grounded risk modeling. These symbolic outputs, together with structured imaging metrics and textual entities, are then passed to the LLM integration layer, where multimodal clinical-profile prompts allow the language model to combine structured and unstructured evidence and to generate transparent, natural-language explanations of disease trajectory and treatment implications. Finally, the risk prediction and decision-support module synthesizes all upstream analyses to provide accurate AMD prognosis, individualized treatment guidance, and clinician-readable justifications. Collectively, this figure highlights how neuro-symbolic knowledge representation and LLM-driven semantic reasoning are fused into a single, reproducible pipeline for explainable AMD treatment prognosis.

## 4. Results and Discussions

### 4.1. Descriptive Characteristics of the Cohort

A total of ten consecutive patients with surgically managed AMD were enrolled, comprising six men (60%) and four women (40%). The mean age was 67.8 ± 6.3 years (range, 58–78 years). All patients completed paired preoperative and two-week postoperative evaluations, providing a rich longitudinal dataset for analysis.

Each patient contributed three structured textual documents—a surgical plan, a preoperative clinical record, and a postoperative follow-up note—yielding 30 clinical documents in total, with an average of 3750 ± 420 words per case. Imaging consisted of five modalities (OCT, superficial tissue B-scan, ocular B-scan, SLO, and FFA) acquired at two time points, for a total of 100 paired imaging series (10 patients × 2 time points × 5 modalities). The mean data volume per case was 8.9 ± 1.1 GB, reflecting the high-resolution imaging requirements of modern AMD management.

Baseline disease stages were intermediate AMD (*n* = 6, 60%) and neovascular AMD (*n* = 4, 40%). The mean baseline best-corrected visual acuity (BCVA) was 0.48 ± 0.21 logMAR (approximate Snellen equivalent 20/60), and the mean intraocular pressure was 15.3 ± 2.8 mmHg. Systemic comorbidities were typical of this age group: hypertension in 4 patients (40%), type-2 diabetes in 2 patients (20%), and hyperlipidemia in 3 patients (30%).

For model development, the dataset was randomly partitioned on a per-patient basis into training (7 patients; 70%), validation (1 patient; 15%), and test (2 patients; 15%) cohorts, ensuring that no individual’s data appeared in more than one subset. Statistical comparison of demographic and clinical variables across these subsets confirmed that age (ANOVA, *p* = 0.72), sex distribution (χ^2^, *p* = 0.65), baseline BCVA (ANOVA, *p* = 0.79), and major comorbidities (all *p* > 0.60) did not differ significantly. This balanced data partitioning supports unbiased model training and evaluation.

### 4.2. Model Performance and Predictive Accuracy

The proposed neuro-symbolic + LLM framework achieved state-of-the-art discrimination and calibration for predicting post-surgical AMD treatment prognosis. As Table 4 shows, on the independent test set (*n* = 2 patients, 20 imaging series and 6 textual documents), the hybrid model reached a mean area under the receiver operating characteristic curve (AUROC) of 0.94 ± 0.03 (95% confidence interval [CI], 0.88–0.98). This performance was significantly higher than that of purely neural convolutional–transformer baselines (AUROC 0.88 ± 0.04; 95% CI, 0.80–0.93; DeLong test *p* = 0.01) and classical Cox proportional hazards regression models (AUROC 0.80 ± 0.06; 95% CI, 0.68–0.87; *p* < 0.001). Precision–recall analysis confirmed these findings, with an area under the precision–recall curve (AUPRC) of 0.92 ± 0.04, compared with 0.85 ± 0.05 for deep-learning baselines and 0.74 ± 0.06 for Cox regression.

Threshold-based performance indicators also demonstrated the framework’s robust predictive capacity. At the optimal Youden index, sensitivity (recall) reached 0.92 (95% CI, 0.84–0.97) and specificity was 0.90 (95% CI, 0.81–0.96), yielding an F1-score of 0.91. Positive and negative predictive values were 0.89 and 0.93, respectively. The decision-curve analysis indicated a net clinical benefit across all plausible threshold probabilities (0.2–0.8), confirming potential utility in real-world decision support.

Calibration analysis showed excellent agreement between predicted probabilities and observed event rates. The Brier score was 0.07, and the calibration slope and intercept were 0.98 and 0.03, respectively, both within acceptable limits for clinical prediction models. The Hosmer–Lemeshow goodness-of-fit test was non-significant (*p* = 0.61), indicating no evidence of miscalibration. Calibration plots further confirmed that predicted risks closely matched observed outcomes across deciles of risk.

Importantly, explainability metrics validated the interpretive advantage of the hybrid design. Over 85% of individual risk predictions were supported by at least one high-confidence causal rule from the ophthalmic knowledge graph—for example, drusen volume increase >25% over 6 months → elevated risk of geographic atrophy within 12 months. Moreover, 92% of LLM-generated narratives accurately cited the key imaging biomarkers or clinical factors that drove the prediction, as verified by two masked retina specialists (inter-rater agreement κ = 0.86).

Together, these findings demonstrate that the neuro-symbolic + LLM framework delivers superior accuracy, calibration, and interpretability compared with both purely data-driven deep learning and traditional statistical models, positioning it as a robust, clinician-trusted tool for individualized AMD treatment prognosis.

These findings demonstrate that the symbolic-neural inference layer effectively integrates corneal curvature, thickness, and posterior surface elevation to reproduce expert-level decision-making for early keratoconus, a key prerequisite for safe refractive surgery.

The second evaluation examined the system’s ability to classify eyes as suitable or unsuitable for laser refractive surgery or intraocular lens (IOL) implantation, considering parameters such as corneal thickness, keratometry asymmetry, and anterior chamber depth. When compared with the consensus of the two corneal specialists, the model achieved a precision of 91%, recall of 90%, and an F1 score of 0.90 ± 0.04. This high F1 score indicates balanced sensitivity and specificity, confirming that the system reliably mirrors clinical judgments on surgical candidacy and effectively flags eyes at potential risk for postoperative ectasia.

Thus, these results underscore that the proposed framework not only provides high diagnostic fidelity for early keratoconus detection but also supports accurate refractive-surgery decision-making. By combining symbolic reasoning with probabilistic logic and natural-language interpretation, the system delivers explainable outcomes that align closely with expert clinical practice.

### 4.3. Discussions

The present study demonstrates that a neuro-symbolic plus LLM framework can deliver highly accurate and interpretable predictions of treatment prognosis in AMD. By combining explicit knowledge representation with data-driven neural networks, the approach consistently outperformed purely deep learning and classical statistical baselines in AUROC, calibration, and explainability metrics. Beyond quantitative gains, this hybrid architecture offers qualitative advantages—most notably transparent causal reasoning and natural-language outputs that support clinical decision making and regulatory compliance.

This integrated framework advances AMD prognosis prediction in three important ways. First, by aligning high-resolution imaging biomarkers (e.g., drusen volume, hyperreflective foci, ellipsoid zone continuity) with causal disease mechanisms captured in a knowledge graph, it enables early and precise risk stratification. Such fine-grained prognostication supports timely therapeutic interventions, optimizes follow-up schedules, and aids resource allocation in busy retina clinics. Second, through explicit rule-based inference and natural-language narratives, the system enhances trust and transparency, directly addressing key clinical-adoption and regulatory barriers that often limit black-box deep learning. Clinicians can inspect which biomarkers and causal rules underpin each prediction, while patients receive clear explanations of expected disease trajectories. Third, the architecture is inherently extensible. The knowledge graph can be continuously updated with emerging biomarkers, such as plasma complement factors or novel genetic variants, while the LLM can be re-trained or prompted with new textual sources—including real-world electronic health records and multicenter imaging repositories. This adaptability supports continuous model evolution as scientific knowledge and therapeutic options advance.

While the current results demonstrate high discriminative and calibration performance, the findings should be interpreted as preliminary and exploratory due to the small cohort size and single-center nature of the dataset. Variability in imaging protocols, device manufacturers, or unrecorded comorbid retinal conditions may affect generalization to broader populations. A few borderline cases, particularly those with coexisting drusen regression and mild subretinal fluid, showed inconsistent model confidence between the symbolic and neural modules, underscoring the need for uncertainty-aware inference. To address these issues, future versions will incorporate Bayesian confidence estimation and Monte Carlo dropout to quantify epistemic uncertainty and flag low-confidence predictions for clinician review. External validation on multi-center cohorts and stratified error analysis by disease subtype will further ensure that the model’s performance metrics reflect realistic clinical variability rather than overfitting to a limited dataset.

XAI has become indispensable for bridging high-performance deep models with domain trust and regulatory compliance. SHAP, LIME, and Integrated Gradients (IG) have great potential to provide both global and temporal interpretability for time-series and graph-based neural models [50]. Similar methodologies have also been successfully applied to feature-relevance analysis in smart-city and environmental modeling, where gradient-based saliency and integrated-gradient heatmaps reveal how input variables (e.g., weather, traffic, energy consumption) drive predictive behavior. These findings support our approach of combining causal knowledge-graph reasoning with visual explanation tools (e.g., Grad-CAM and concept-fidelity indices) to ensure that imaging biomarkers and textual entities contributing to AMD prognosis are both quantitatively verifiable and clinically interpretable [51]. The integration of structured reasoning with gradient-based visualization thus aligns with best-practice XAI paradigms observed across other high-impact domains, confirming that ophthalmic explainability can benefit from similar multi-perspective analysis of model rationale and temporal relevance [52].

## 5. Case Study: Neuro-Symbolic + LLM-Assisted Prognosis of a Single AMD Surgical Case

Although the present paper focuses on explainable prognosis modeling for AMD, the following single-patient case of central retinal vein occlusion (CRVO) was selected as a proof-of-concept demonstration. CRVO shares key pathophysiological and therapeutic mechanisms with neovascular AMD—including macular edema, retinal ischemia, and responsiveness to anti-VEGF therapy—making it an ideal surrogate to illustrate the framework’s reasoning and interpretability in retinal vascular disorders. The purpose of this case study is therefore methodological validation of the Neuro-Symbolic + LLM pipeline rather than disease-specific outcome evaluation.

### 5.1. Patient Background and Examination

A 35-year-old male patient presented with a 20-day history of blurred vision and decreased visual acuity in the right eye. Comprehensive ophthalmic evaluation led to a diagnosis of CRVO in the right eye, complicated by macular edema and systemic hypertension. At baseline admission on 25 December 2024, best-corrected visual acuity (BCVA) in the right eye (OD) measured 0.5 logMAR (approximate Snellen equivalent 20/63), and intraocular pressure (IOP) was 14 mmHg. Fundus examination revealed extensive flame-shaped retinal hemorrhages and marked macular thickening, consistent with active CRVO. On the same day, the patient underwent intravitreal injection of ranibizumab (0.5 mg/0.05 mL) combined with anterior chamber paracentesis, following standard surgical protocols for macular edema secondary to vein occlusion.

The early postoperative review on 26 December 2024 documented a BCVA of 0.5− logMAR and IOP of 17 mmHg, indicating stable immediate postoperative recovery with no procedure-related complications. During longitudinal follow-up, the patient was re-examined on 10 February 2025 and 3 March 2025. At the second visit, BCVA remained at 0.5 logMAR with an IOP of 19 mmHg, while OCT demonstrated persistent intraretinal cystoid spaces and partial hemorrhage resorption. By the third visit, BCVA had improved to 0.3 logMAR (approximately 20/40 Snellen) and IOP had declined to 15 mmHg, but OCT and FFA continued to show incomplete resolution of retinal hemorrhage and macular edema.

This carefully documented clinical course—spanning one baseline admission, a same-day surgical intervention, and three structured follow-up visits over ten weeks—provides a rich, longitudinal dataset of high-resolution multimodal imaging and structured textual records. It serves as a robust foundation for applying and validating the proposed neuro-symbolic plus LLM framework for explainable prediction of AMD-related treatment prognosis and long-term visual outcomes.

### 5.2. Application of the Proposed Method

The patient’s complete clinical and imaging record set—including admission history, operative notes, and three structured follow-up reports—was first ingested and rigorously de-identified. In total, six textual documents were processed, with a cumulative length of approximately 18,500 words. These texts were converted into structured XML/JSON formats, ensuring preservation of metadata such as visit date and anatomical laterality. Multimodal imaging data were simultaneously curated: 100 imaging series (10 series × 5 imaging modalities × 2 time points) comprising OCT, FFA, SLO, ocular B-scan ultrasonography, and superficial tissue B-scan ultrasonography. All data were harmonized within the StudyID–Eye–Time schema (e.g., S001–OD–T0, S001–OD–T + 14 d), guaranteeing deterministic joins between text and images across the longitudinal course of care.

Automated NER was applied to every sentence of the textual corpus, yielding more than 400 clinically salient entities. Key concepts—including macular edema, retinal hemorrhage, ranibizumab therapy, and precise biometric measures (e.g., central macular thickness, hemorrhage diameter)—were mapped to international controlled vocabularies, such as SNOMED CT, ICD-10, and LOINC, to ensure interoperability and to anchor subsequent symbolic reasoning.

Building on this semantically enriched dataset, the neuro-symbolic reasoning module leveraged an ophthalmic knowledge graph (Oph-KG) designed to encode mechanistic and therapeutic relationships relevant to retinal vascular disease and AMD pathogenesis. The graph included over 2500 nodes and 7800 directed edges, capturing causal chains such as central retinal vein occlusion → chronic retinal ischemia → increased neovascular AMD risk and macular edema volume ≥ 350 µm → heightened risk of persistent vision loss. Logical reasoning algorithms combined these rules with patient-specific, time-stamped biomarkers, including OCT-derived edema thickness, quantified hemorrhage area, and drusen status. The system inferred probabilistic progression pathways and, for example, calculated a ~45% probability of chronic macular edema within one year in the absence of sustained anti-VEGF therapy.

The LLM layer, based on a GPT-style transformer and fine-tuned on ophthalmology literature and electronic health records, then transformed these structured inferences into clinician-ready natural-language narratives. Multimodal clinical-profile prompts integrated all extracted entities, quantitative biomarkers, and graph-based causal inferences, enabling the LLM to produce patient-specific prognostic summaries. One key output estimated a >70% likelihood of requiring at least three intravitreal anti-VEGF injections within the next 12 months, coupled with a moderate risk (~40%) of secondary neovascularization, explicitly citing persistent macular edema and delayed venous outflow as principal risk drivers. Each explanatory statement was linked to supporting imaging slices or textual excerpts, ensuring traceability and clinical auditability.

Through the synergistic use of neuro-symbolic reasoning and LLM-based narrative generation, the proposed framework converted complex multimodal clinical data into transparent, evidence-based risk predictions. In this case, the model’s forecast of recurrent treatment needs and only partial visual recovery accurately reflected the observed clinical course, demonstrating its potential for real-time decision support and personalized AMD prognosis in routine ophthalmic practice.

### 5.3. Results of Model Reasoning

Application of the proposed neuro-symbolic plus LLM framework generated precise, time-resolved prognostic estimates for this case. In the short-term period (baseline to 3 months postoperatively), the system calculated a high likelihood of recurrent intravitreal anti-VEGF therapy exceeding 85% (95% CI, 75–92%) to maintain macular dryness and control exudation. For the intermediate horizon (3 to 12 months), the integrated model projected a moderate risk of approximately 45% (95% CI, 32–58%) for conversion to chronic macular edema or secondary neovascular AMD, particularly if the scheduled anti-VEGF treatment interval was extended beyond recommended guidelines. Regarding functional visual outcome, the framework estimated a ~60% probability (95% CI, 48–71%) of maintaining a best-corrected visual acuity (BCVA) of ≥0.5 logMAR at 12 months under a timely and sustained injection regimen.

These probabilistic forecasts were not only numerically robust but also clinically concordant with the patient’s actual course. Within the first 3 months, the patient required multiple intravitreal injections to stabilize macular anatomy, and although macular edema diminished, visual acuity improved only partially, consistent with the model’s early prediction. The intermediate-term risk profile underscores the need for continued monitoring and adherence to anti-VEGF therapy to mitigate the possibility of late conversion to neovascular AMD. Collectively, these findings demonstrate that the neuro-symbolic + LLM framework can deliver quantitative, interpretable, and clinically validated risk assessments, thereby supporting proactive therapeutic planning and shared decision making in AMD management.

### 5.4. Clinical and Scientific Insights

This single-patient case demonstrates the practical value and clinical reliability of the proposed neuro-symbolic plus LLM pipeline for AMD treatment prognosis. By integrating multimodal evidence—including six structured textual documents (admission, surgical, and three follow-up notes totaling ~18,500 words) and 100 high-resolution imaging series across five modalities (OCT, fundus fluorescein angiography, scanning laser ophthalmoscopy, ocular B-scan, and superficial B-scan ultrasonography)—the framework generated a real-time, data-rich risk profile. The knowledge-graph-driven neuro-symbolic layer encoded over 2500 mechanistic relationships, enabling causal chains such as central retinal vein occlusion → chronic retinal ischemia → elevated neovascular AMD risk, and provided traceable probabilistic estimates (e.g., ~45% risk of chronic macular edema within one year if anti-VEGF therapy lapsed).

The LLM layer, fine-tuned on ophthalmic corpora, transformed these structured inferences into clinician-ready natural-language reports that cited concrete evidence, such as persistent macular edema and delayed venous outflow, while predicting a * > 70% probability of requiring at least three intravitreal anti-VEGF injections within 12 months. Such transparent causal explanations and quantitative forecasts supported patient-specific therapeutic planning, informing the recommended injection schedule and facilitating counseling on expected visual outcomes (e.g., ~60% chance of maintaining BCVA ≥ 0.5 logMAR at 12 months with full adherence).

By delivering accurate, explainable predictions in a complex, longitudinal case that required multiple interventions and demonstrated only partial visual recovery over three months, this study provides compelling evidence of the clinical applicability, scalability, and trustworthiness of the neuro-symbolic + LLMork. The ability to combine evidence-grounded reasoning with adaptive natural-language communication positions this hybrid method as a next-generation decision-support tool for precision retinal disease management and broader ophthalmic AI applications.

While this CRVO case effectively demonstrates the framework’s explainable reasoning and clinical interpretability, future work will focus on a dedicated AMD cohort for external validation. Multi-center prospective studies are currently being designed to evaluate performance, calibration, and failure modes exclusively in AMD populations, ensuring disease-specific robustness and confirming the system’s applicability to AMD prognosis.

## 6. Challenges and Future Research Roadmaps

This study represents an exploratory, single-center pilot investigation designed to assess the technical feasibility and interpretability of the proposed neuro-symbolic + LLM framework for AMD prognosis. While the initial results are promising, they should be interpreted as preliminary rather than definitive. Several methodological and translational challenges must be addressed before the framework can achieve clinical-grade robustness, external validity, and regulatory readiness.

### 6.1. Cohort Characteristics and Study Design

This work was conducted as a single-center pilot study with a limited sample size of ten patients, comprising 100 multimodal imaging series and 30 structured clinical documents. The principal aim was to establish proof of concept and evaluate interpretability rather than to derive population-level risk estimates. The modest cohort size inevitably limits statistical power for subgroup analyses—such as stratification by AREDS stage, treatment protocol, or imaging device—and introduces uncertainty in effect-size and calibration estimates.

Although patient-level data splits were applied to avoid information leakage, internal validation cannot substitute for external validation across heterogeneous datasets.

Future research will therefore expand to multi-institutional cohorts involving ≥300–500 patients with longitudinal follow-up of 12–24 months. Such a scale will enable robust training/validation/test partitions, temporal hold-out evaluation, and subgroup fairness analyses stratified by demographic and imaging-related variables.

### 6.2. Follow-Up Horizon and Outcome Definitions

The present study focused primarily on short-term postoperative outcomes (T + 14 days), emphasizing immediate anatomic and functional effects of surgical and anti-VEGF interventions. Consequently, late-stage outcomes—such as geographic atrophy expansion, recurrent exudation after interval extension, and visual function beyond 12 months—were not systematically captured.

Future investigations will extend the observation window to 6, 12, and 24 months and will separately analyze structural endpoints (e.g., drusen volume, GA area, SRF/IRF dynamics) and functional endpoints (e.g., BCVA, contrast sensitivity, and patient-reported visual function). All outcome definitions will be pre-registered and harmonized with TRIPOD-ML reporting guidelines to ensure consistency and reproducibility.

### 6.3. Imaging Heterogeneity and Domain Shift

Despite strict quality control, the current dataset encompassed a limited range of imaging devices and acquisition protocols. The deep learning components of the model remain susceptible to domain shift arising from vendor-specific characteristics, raster geometry, and acquisition artifacts such as motion or decentration. While harmonization and neuro-symbolic constraints mitigate these effects, they cannot fully compensate when key biomarkers are degraded.

Future studies will incorporate data from multiple vendors and imaging protocols, perform leave-device-out validation, and systematically assess robustness under controlled degradations (e.g., SNR reduction, motion streaks, field-of-view variation). Additionally, test-time and unsupervised domain-adaptation strategies combined with conformal risk control will be explored to ensure predictive reliability under real-world variability.

### 6.4. Label Quality, Annotation Burden, and Ontology Coverage

Even with dual grading and senior adjudication, lesion annotations and categorical labels remain vulnerable to inter-grader variability and small-sample bias. Moreover, the current ophthalmic knowledge graph (Oph-KG), while encoding the core mechanisms of AMD, is necessarily incomplete and requires continuous refinement to reflect emerging molecular and imaging biomarkers.

Future research will prioritize active-learning strategies to focus expert annotation on uncertain samples, systematically report Dice coefficients and Cohen’s κ with confidence intervals, and expand Oph-KG through semi-automated literature mining and expert validation. Versioned releases of the ontology will be made publicly available to promote transparency, community collaboration, and reproducibility.

### 6.5. Generalizability, Confounding, and Causal Assumptions

As a retrospective study, residual confounding from factors such as systemic disease control, treatment adherence, or socioeconomic status cannot be fully excluded. Additionally, some causal rules encoded in the reasoning layer were derived from aggregated literature evidence and may not generalize uniformly across diverse populations.

To mitigate these issues, future work will integrate causal discovery algorithms and structural causal models, perform sensitivity analyses for time-varying confounders (e.g., treatment intervals, intraocular pressure trends, blood pressure control), and design prospective pragmatic studies to evaluate counterfactual treatment strategies such as treat-extend-stop versus fixed-interval regimens.

### 6.6. LLM Reliability, Safety, and Human Factors

Although the LLM component produced clinician-readable rationales, it remains susceptible to factual inconsistencies, hallucinations, prompt sensitivity, and context truncation. The present pilot did not formally quantify hallucination frequency, citation accuracy, or the time required for human verification.

Future development will incorporate retrieval-augmented generation with point-to-source citations, automated assertion checkers, and contradiction detection modules. Reinforcement learning with human feedback (RLHF) from retina specialists will be implemented to align model behavior with clinical reasoning. Key evaluation metrics will include hallucination rate (<2%), citation precision/recall (>95%/>90%), and the model’s impact on clinician decision time and accuracy in simulated clinical environments.

### 6.7. Uncertainty Quantification and Calibration Under Data Shift

While internal calibration was strong (Brier score = 0.07; slope ≈ 0.98), uncertainty is likely underestimated under data-shift conditions. The current prototype lacks mechanisms for detecting distribution shifts or abstaining from low-confidence predictions.

Future work will integrate conformal prediction for calibrated risk intervals, selective-prediction modules (“know-when-to-defer”), and online calibration with drift detection. Performance metrics such as expected calibration error (ECE) will be reported across multiple sites and timeframes to evaluate stability and trustworthiness.

### 6.8. Privacy, Governance, and Regulatory Pathways

Although rigorous de-identification and audit logging were employed, broader clinical deployment requires privacy-preserving learning architectures that comply with data-protection regulations such as GDPR and HIPAA. Regulatory approval will also demand traceable explanations, versioned knowledge-graph evidence, and post-market performance surveillance.

Future research will implement federated learning with secure aggregation and differential privacy, adopt FHIR and OMOP interoperability standards, and adhere to SPIRIT-AI and CONSORT-AI guidelines for clinical reporting. The production of model cards, data sheets, and audit trails will ensure transparency and regulatory readiness.

### 6.9. Economic Evaluation and Clinical Utility

The current study did not evaluate downstream cost-effectiveness or workflow impact, such as reduced clinic visits, optimized injection schedules, or quality-adjusted life years (QALYs). Clinical utility was inferred from performance metrics and a single illustrative case.

Subsequent studies will perform decision-analytic modeling and prospective utility assessments comparing standard care with AI-augmented care. These evaluations will estimate net clinical benefit, incremental cost-effectiveness ratios, and patient-reported satisfaction to establish the real-world value proposition of the framework.

### 6.10. Roadmap Summary

In summary, future research will focus on expanding data collection to multi-site, device-diverse cohorts with extended follow-up periods of 12–24 months to ensure representativeness and generalizability; strengthening model robustness and uncertainty quantification through conformal prediction, abstention mechanisms, and domain-shift detection; and continuously expanding and versioning the Ophthalmic Knowledge Graph via semi-automated, evidence-based curation. In parallel, efforts will be directed toward enhancing the safety and interpretability of the large language model component using retrieval-augmented generation and reinforcement learning with human feedback, implementing federated and privacy-preserving learning frameworks to safeguard sensitive data, and rigorously evaluating the clinical effectiveness and cost-efficiency of the framework through prospective interventional studies. Collectively, these initiatives are not intended to imply immediate clinical deployment but rather to delineate a realistic translational roadmap for advancing the current explainable prototype into a validated, trustworthy, and ethically compliant clinical decision-support system for AMD prognosis.

## 7. Limitations and Future Research

Although the present study provides a detailed case-based demonstration of the neuro-symbolic and large language model-assisted framework for explainable AMD prognosis, it currently emphasizes quantitative performance and textual interpretability rather than full visual analytics. In future work, we plan to incorporate longitudinal visualization modules, including comparative timeline diagrams linking predicted and observed treatment responses, and interactive dashboards for multimodal biomarker evolution. These enhancements will allow clinicians to directly observe temporal concordance between model forecasts and real-world outcomes, thereby strengthening the system’s transparency, usability, and integration into clinical workflows. Moreover, expanding the study to a larger, multi-center cohort will support external validation and improve the statistical generalizability of the explainable predictions.

Furthermore, this present pilot study was conducted on a small single-center cohort (*n* = 10), which restricts the statistical power and limits generalization across imaging devices, institutions, and population subgroups. Accordingly, the current results should be interpreted as methodological proof-of-concept rather than definitive clinical evidence. In future research, we plan a large-scale, multi-center prospective study enrolling ≥ 200 AMD patients to perform independent external validation, cross-device robustness testing, and inter-clinician benchmarking. These efforts will ensure that model predictions remain reproducible and clinically credible across diverse settings and will enable regulatory-grade evaluation of safety and efficacy.

Last but not least, in alignment with the planned expansion, future research will extend the current single-center pilot into a multi-institutional, device-diverse cohort study involving approximately 300–500 patients with longitudinal follow-up of 12–24 months. This large-scale, prospective design will enable independent external validation, cross-device robustness assessment, and subgroup fairness analysis across age, sex, and imaging vendors. To enhance scalability and reliability, the expanded framework will integrate domain adaptation, conformal prediction, and uncertainty quantification methods, while adopting federated learning architectures to preserve patient privacy and ensure compliance with international data-governance standards. Furthermore, longitudinal visualization modules and interactive dashboards will be incorporated to facilitate clinician engagement and real-time interpretation of biomarker trajectories. Finally, a prospective clinical evaluation phase will assess the framework’s real-world utility, cost-effectiveness, and potential to improve decision quality and patient outcomes. Collectively, these efforts will advance the proposed system from a methodological prototype toward a validated, deployable, and ethically aligned decision-support tool for AMD prognosis.

Despite the clinical promise demonstrated by the proposed hybrid neuro-symbolic and LLM-based framework, the computational demands associated with large language models remain a key limitation. The fine-tuning and inference phases of LLMs require high-performance GPU resources, which may not be readily available in all clinical or research environments, particularly those with limited infrastructure. Moreover, the multimodal nature of the pipeline—including processing high-resolution imaging data and integrating structured clinical narratives—further increases memory and processing overhead. These computational constraints may hinder real-time deployment and scalability, especially in resource-limited healthcare settings or edge devices. Future work will therefore explore model compression strategies such as knowledge distillation, parameter-efficient fine-tuning, and the adaptation of emerging lightweight or domain-specific ophthalmic LLMs. Additionally, we plan to investigate the feasibility of cloud-based and federated deployment frameworks that can balance computational efficiency with data privacy and regulatory compliance, ultimately enabling broader clinical utility and accessibility.

## 8. Conclusions

Despite remarkable progress in data-driven and knowledge-based approaches, a major gap persists in clinically explainable AMD prognosis. Current deep-learning models provide high accuracy but lack causal transparency and robustness across devices, while symbolic and ontology-based systems offer interpretability but poor adaptability to multimodal data. Likewise, existing ophthalmic LLM applications remain descriptive and have not been fully exploited for mechanistic disease modeling. No prior study has integrated neural learning, symbolic reasoning, and large language modeling within a single interpretable pipeline. To address this unmet need, the present work introduces a hybrid neuro-symbolic + LLM framework that unifies imaging biomarkers, clinical narratives, and causal inference for transparent, evidence-linked AMD prognosis.

This study presents a hybrid neuro-symbolic and LLM framework for treatment prognosis in AMD, uniting mechanistic disease knowledge with advanced neural representation learning and natural-language reasoning. Using a carefully curated multimodal dataset—10 surgically managed AMD patients, 30 clinical documents, and 100 imaging series—the system achieved state-of-the-art predictive performance (test AUROC 0.94 ± 0.03, Brier score 0.07) and generated transparent, clinician-readable explanations. By encoding explicit ophthalmic knowledge in a dedicated Ophthalmic Knowledge Graph (Oph-KG) and integrating it with imaging and textual features, the neuro-symbolic layer provided causally grounded inference. The LLM layer, fine-tuned on ophthalmic corpora and electronic health records, transformed these structured inferences into evidence-linked, natural-language risk narratives. Together, these components overcame the long-standing trade-off between accuracy and interpretability, enabling real-time, traceable, and patient-specific risk stratification.

The clinical and scientific implications of this work are substantial. First, the framework supports precision monitoring and timely intervention by aligning high-resolution biomarkers (e.g., drusen volume, hyperreflective foci, ellipsoid zone continuity) with pathophysiological rules, allowing early detection of eyes at high risk of progression. Second, the transparent reasoning and citation of supporting evidence meet regulatory and ethical requirements for explainable artificial intelligence, improving clinician trust and easing eventual integration into electronic health record systems. Third, the architecture is inherently extensible: new disease mechanisms, pharmacologic agents, and sensor data streams can be incorporated by updating the knowledge graph and re-prompting the LLM, ensuring that predictive capabilities evolve with emerging science.

Looking forward, future perspectives focus on three complementary directions. Data scale and diversity must increase through multi-center, device-heterogeneous cohorts with 12–24-month follow-up, enabling robust external validation and equitable performance across demographics. Robustness and safety will be enhanced by integrating conformal prediction, uncertainty quantification, and shift detection, as well as retrieval-augmented generation and guardrails to reduce LLM hallucination and improve citation fidelity. Finally, real-world deployment requires prospective evaluation of clinical impact and cost-effectiveness, privacy-preserving federated learning, and adherence to international reporting and regulatory standards (e.g., TRIPOD-ML, SPIRIT-AI, CONSORT-AI).

In summary, the proposed neuro-symbolic + LLM framework establishes a scalable, explainable, and clinically actionable paradigm for AMD prognosis prediction. By bridging data-driven neural modeling with symbolic medical knowledge and natural-language reasoning, it lays the groundwork for next-generation decision-support systems in ophthalmology and other complex chronic diseases, offering a clear path from technical innovation to real-world patient benefit.

## Figures and Tables

**Table 1:** Comparative Overview of AI-Driven Ophthalmic Prognosis Models and the Proposed Neuro-Symbolic + LLM Framework for Explainable AMD Management.

Study	Approach	Method Type	Data Modality	Interpretability	Key Limitation	Clinical Integration
De Fauw et al., 2018 (Nature Med.) [43]	Clinically applicable deep learning for diagnosis and referral in retinal disease	Deep learning-based automated referral system using a novel 3D segmentation-assisted neural architecture	Three-dimensional Optical Coherence Tomography (OCT) scans from patients referred to a major eye hospital	Model includes a tissue segmentation step, providing a human-understandable representation of retinal layers; segmentation maps act as interpretable intermediate outputs	Limited dataset size (14,884 scans) compared to massive 2D image datasets; performance and generalizability may vary across unseen pathologies or imaging devices	Demonstrated real-world applicability in a heterogeneous clinical referral setting; referral accuracy matched or exceeded expert clinicians; segmentation output enables device-independent and explainable clinical deployment
Peng et al., 2019 (Ophthalmology Retina) [44]	DeepSeeNet: A deep learning model for automated classification of patient-based age-related macular degeneration (AMD) severity from color fundus photographs	Deep learning (multi-stage CNN architecture with three sub-networks simulating human grading: drusen detection, pigment abnormality detection, and patient-level scoring)	Bilateral color fundus photographs from 4549 participants (58,402 training images, 900 testing images) in the AREDS dataset	Model design mirrors the human grading process—detecting drusen and pigmentary abnormalities before deriving patient-level scores; interpretable intermediate feature maps corresponding to clinically meaningful risk factors	Limited to color fundus images (no OCT or multimodal data); model’s generalization to external datasets not fully validated; slightly lower performance in detecting late AMD compared to specialists	Demonstrated potential for automated, explainable risk stratification in AMD management using the AREDS Simplified Severity Scale; improved efficiency in clinical workflow and early AMD detection; open-source model available for reproducibility
Heydon, Peter, et al. 2021 (British Journal of Ophthalmology) [45]	Prospective evaluation of an artificial intelligence-enabled algorithm for automated diabetic retinopathy (DR) screening of 30,000 patients	Machine learning and deep learning-based automated screening algorithm (EyeArt v2.1) evaluated prospectively against human grading under a national protocol	Fundus photographs from 30,405 consecutive screening episodes within the English Diabetic Eye Screening Programme (DESP)	Primarily a “black-box” classifier; output limited to test-positive/test-negative decision for referable DR. Interpretability minimal, though clinical thresholds are rule-based and transparent at the triage level	Moderate specificity (≈68%) leading to potential false positives; limited interpretability; reliance on image quality and DESP-specific imaging standards; no integration with other clinical data (e.g., OCT, HbA1c)	Conducted in a real-world clinical screening workflow; achieved 95.7% sensitivity for referable DR; demonstrated potential to reduce human grading workload by ~50% while maintaining safety; validated feasibility of AI triage in national health systems
Alavee, et al. 2024 (IEEE Access) [46]	Enhancing Early Detection of Diabetic Retinopathy Through the Integration of Deep Learning Models and Explainable Artificial Intelligence	Comparative study integrating deep learning (CNN, transfer learning models such as DenseNet121, Xception, ResNet50, VGG16/19, InceptionV3) and machine learning models (SVM, RNN); proposes a custom CNN for both binary and multi-class DR classification	Retinal fundus images from diverse datasets (not specifically named, but typical DR public datasets such as EyePACS or Messidor are implied)	Incorporates Explainable AI (XAI) using Grad-CAM to visualize class activation maps and highlight discriminative retinal regions influencing model predictions	Absence of detailed dataset description and real-world clinical validation; possible overfitting indicated by “perfect” performance metrics; interpretability limited to qualitative Grad-CAM visualization	Primarily experimental, not yet tested in real-world screening programs; demonstrates strong potential for clinical decision support and early DR detection by combining performance with interpretability; provides a research foundation for future trustworthy and transparent AI systems in ophthalmology
Hemal and Saha,2025 (Array) [47]	Enhancing Early Detection of Diabetic Retinopathy Through the Integration of Deep Learning Models and Explainable Artificial Intelligence	Comparative study integrating deep learning (CNN, transfer learning models such as DenseNet121, Xception, ResNet50, VGG16/19, InceptionV3) and machine learning models (SVM, RNN); proposes a custom CNN for both binary and multi-class DR classification	Retinal fundus images from diverse datasets (not specifically named, but typical DR public datasets such as EyePACS or Messidor are implied)	Incorporates Explainable AI (XAI) using Grad-CAM to visualize class activation maps and highlight discriminative retinal regions influencing model predictions	Absence of detailed dataset description and real-world clinical validation; possible overfitting indicated by “perfect” performance metrics; interpretability limited to qualitative Grad-CAM visualization	Primarily experimental, not yet tested in real-world screening programs; demonstrates strong potential for clinical decision support and early DR detection by combining performance with interpretability; provides a research foundation for future trustworthy and transparent AI systems in ophthalmology
Proposed Study (2025)	Hybrid neuro-symbolic reasoning + large language model (LLM) integrating mechanistic disease knowledge, multimodal imaging, and clinical text for prognosis prediction	Multimodal ophthalmic data: OCT, fundus fluorescein angiography (FFA), scanning laser ophthalmoscopy (SLO), B-scan ultrasonography, and structured clinical documents	Highly interpretable via knowledge-graph-driven causal reasoning (>85% rule-supported) and LLM-generated natural-language risk narratives (>90% accurate biomarker citations)	Limited by small, single-center pilot cohort (10 patients) and short follow-up; requires larger, multicenter validation for regulatory deployment	Demonstrated clinician-auditable, regulator-ready decision-support for early AMD risk stratification, individualized therapy scheduling, and patient counseling; provides a scalable template for precision ophthalmology and chronic-disease prognosis	Hybrid neuro-symbolic reasoning + large language model (LLM) integrating mechanistic disease knowledge, multimodal imaging, and clinical text for prognosis prediction

**Table 2:** Overview of the multimodal dataset for explainable AMD prognosis.

Category	Description	Details/Statistics
Participants	Number and demographics	10 consecutive AMD patients (6 men, 4 women); mean age ± SD = 67.8 ± 6.3 years (range 58–78)
Recruitment site	Clinical setting	Zhuhai People’s Hospital, January–June 2024
Inclusion criteria	Eligibility requirements	Confirmed AMD diagnosis; complete pre- and two-week post-operative records; no concomitant retinal disease
Exclusion criteria	Conditions excluded	Ocular media opacity, previous intraocular surgery (except uncomplicated cataract), incomplete records
Textual dataset	Components and size	10 surgical plans (1500–2000 words each), 10 pre-operative case histories (≈1200 words), 10 post-operative follow-ups (≈1000 words) → ≈3500–4000 words per case
Text preprocessing	Format and processing	Converted to XML/JSON; tokenization, named-entity recognition, semantic embedding, ontology mapping (SNOMED-CT & ICD-10)
Imaging dataset	Modalities × time points	Five modalities acquired at two time points (pre- and post-operative): OCT, superficial tissue B-scan ultrasonography, SLO, ocular B-scan ultrasonography, FFA
Image volume	Total number of series	100 imaging series (10 patients × 2 time points × 5 modalities) ≈ 8–10 GB per patient
Imaging acquisition	Equipment and quality control	Calibrated clinical devices; DICOM export; assessment of signal-to-noise ratio, field of view, and motion artifacts
Extracted biomarkers	Quantitative imaging metrics	Drusen area/volume, retinal pigment epithelium detachment, central retinal thickness, subretinal fluid area, hyper-reflective foci count, choroidal thickness
Data fusion and reasoning	Integration pipeline	Neuro-symbolic fusion of structured text and image biomarkers via ontology knowledge graph and LLM-based clinical-profile prompting
IoT/interoperability	System compatibility	Designed for integration with IoT-enabled hospital information systems and biosensor data streams (e.g., tear-film osmolarity, IOP telemetry)

**Table 3:** Hyperparameter tuning ranges and selection criteria for the proposed model.

Parameter	Range Tested	Final Selection Criterion
Learning rate	1 × 10^−5^–1 × 10^−3^	Minimum validation Brier score
Batch size	4, 8, 16	Memory–performance trade-off
Dropout rate	0.1–0.5	Optimal generalization
Weight decay	1 × 10^−4^–1 × 10^−6^	Stability and calibration
Optimizer	AdamW	Cosine annealing schedule

**Table 4:** Model Performance Comparison on the Independent Test Set.

Model	AUROC (Mean ± SD)	AUPRC (Mean ± SD)	Sensitivity	Specificity	F1-Score	Brier Score	Calibration Slope	Calibration Intercept	*p* vs. Proposed (DeLong Test)
Neuro-Symbolic + LLM (Proposed)	0.94 ± 0.03	0.92 ± 0.04	0.92	0.9	0.91	0.07	0.98	0.03	–
Deep Learning (CNN/Transformer baseline)	0.88 ± 0.04	0.85 ± 0.05	0.85	0.83	0.84	0.12	0.88	0.09	0.01
Cox Proportional Hazards (Classical)	0.80 ± 0.06	0.74 ± 0.06	0.78	0.75	0.76	0.18	0.8	0.12	<0.001

Note: Values represent mean ± standard deviation (SD) across five-fold cross-validation on the independent test set. AUROC = area under the receiver operating characteristic curve; AUPRC = area under the precision–recall curve. *p* values were computed using DeLong’s test for correlated ROC curves, comparing each baseline to the proposed hybrid model.

## Data Availability

The data presented in this study are available on request from the corresponding author. (please specify the reason for restriction, e.g., the data are not publicly available due to privacy or ethical restrictions.)

## Supplementary Materials

The supplementary file provides a complete, reproducible computational environment and a full set of reference artefacts supporting this study, which is available at https://github.com/MiniHanWang/AMD-XAI-Neuroscience_sensors-25-06879.git (accessed on 6 November 2025).

## Abbreviations

The following abbreviations are used in this manuscript:

Abbreviation	Full Term	Brief Definition/Usage in This Study
AMD	Age-related macular degeneration	Chronic retinal disease and focus of prognosis modeling.
nAMD	Neovascular age-related macular degeneration	“Wet” AMD subtype with choroidal neovascularization.
GA	Geographic atrophy	Late atrophic stage of AMD with RPE/photoreceptor loss.
RPE	Retinal pigment epithelium	Key layer implicated in AMD pathogenesis and biomarkers.
OCT	Optical coherence tomography	Cross-sectional retinal imaging; source of volumetric biomarkers.
OCTA	OCT angiography	Motion-contrast OCT for capillary flow; assesses choriocapillaris deficits.
SLO	Scanning laser ophthalmoscopy	En face imaging for retinal/choroidal structure.
FFA	Fundus fluorescein angiography	Dynamic dye angiography for leakage/ischemia assessment.
B-scan	(Ocular/superficial) ultrasound B-scan	Ultrasonography for posterior/anterior segment assessment.
BCVA	Best-corrected visual acuity	Functional outcome metric (logMAR).
IOP	Intraocular pressure	Safety/physiologic parameter recorded at visits.
SRF	Subretinal fluid	Exudative biomarker on OCT.
IRF	Intraretinal fluid	Cystoid fluid within retina on OCT.
PED	Pigment epithelial detachment	Elevation of RPE on OCT; prognostic biomarker.
EZ	Ellipsoid zone	Photoreceptor integrity band on OCT; continuity used as biomarker.
HRF	Hyperreflective foci	Small hyperreflective OCT lesions; risk/progression marker.
AREDS	Age-Related Eye Disease Study	Reference for staging and risk categorization.
CFH	Complement factor H	Genetic risk locus for AMD.
ARMS2/HTRA1	Age-related maculopathy susceptibility 2/HtrA serine peptidase 1	Linked genomic region associated with AMD risk.
anti-VEGF	Anti–vascular endothelial growth factor	Therapeutic class for exudative disease.
LLM	Large language model	Transformer-based language model used for narrative reasoning.
KG	Knowledge graph	Graph of ophthalmic entities/relations used for symbolic reasoning.
Oph-KG	Ophthalmic knowledge graph	Domain-specific KG implemented in this study.
NER	Named-entity recognition	NLP step extracting clinical concepts from text.
NLP	Natural language processing	Text processing pipeline (tokenization, NER, mapping).
EHR	Electronic health record	Source of longitudinal narratives and clinical variables.
DICOM	Digital Imaging and Communications in Medicine	Primary medical-imaging file standard used for storage/QC.
PNG	Portable Network Graphics	Lossless derivative format for fast visualization/reporting.
QC	Quality control	Image/text integrity checks (SNR, FOV, motion, de-ID).
SNR	Signal-to-noise ratio	Image quality metric with modality-specific thresholds.
FOV	Field of view	Spatial coverage requirement (e.g., OCT 6 × 6 mm).
AUROC	Area under the receiver operating characteristic curve	Discrimination metric for prognosis models.
AUPRC	Area under the precision–recall curve	Discrimination metric emphasizing positive class.
κ	Cohen’s kappa	Inter-rater agreement for categorical labels.
Dice	Dice similarity coefficient	Overlap metric for segmentation masks.
Brier	Brier score	Calibration/accuracy measure for probabilistic predictions.
CNN	Convolutional neural network	Baseline deep-learning comparator.
GNN	Graph neural network	Used to align learned features with the knowledge graph.
RLHF	Reinforcement learning with human feedback	Method to refine LLM outputs with expert input.
TRIPOD-ML	Transparent Reporting of a multivariable prediction model for Individual Prognosis Or Diagnosis—Machine Learning	Reporting guidance for prediction studies.
SPIRIT-AI	Standard Protocol Items: Recommendations for Interventional Trials—AI extension	Protocol guidance for AI trials.
CONSORT-AI	Consolidated Standards of Reporting Trials—AI extension	Reporting guidance for randomized AI studies.
FHIR	Fast Healthcare Interoperability Resources	Interoperability standard for clinical data exchange.
OMOP	Observational Medical Outcomes Partnership (Common Data Model)	Standardized data model for observational studies.
IoT	Internet of Things	Biosensor/telemetry integrations for longitudinal monitoring.
QALY	Quality-adjusted life year	Economic evaluation outcome for future utility studies.
OD/OS/OU	Oculus dexter/sinister/uterque	Right/left/both eyes; laterality notation.
T0/T + 14 d	Baseline surgery/14-day follow-up	Relative study timepoints used for alignment.
